# Patterns of intrinsic capacity trajectory and onset of activities of daily living disability among community-dwelling older adults

**DOI:** 10.7189/jogh.14.04159

**Published:** 2024-10-11

**Authors:** Shan Zhang, Shuqing Wu, Rongrong Guo, Shu Ding, Ying Wu

**Affiliations:** 1 School of Nursing, Capital Medical University, Beijing, China; 2 Department of Cardiology, Sun Yat-Sen Memorial Hospital of Sun Yat-Sen University, Guangzhou, China; 3 Nursing department, Beijing Chaoyang Hospital Affiliated to Capital Medical University, Beijing, China

## Abstract

**Background:**

Global population ageing has brought about new challenges for elderly care. Exploring intrinsic capacity (IC) over time, which is designed as a composite measure of an individual’s physical and mental capabilities, is essential for promoting healthy ageing and preventing dependency, such as that emerging from disability in activities of daily living (ADL). We aimed to identify and examine the differences between classes of IC trajectory and onset of ADL disability.

**Methods:**

We conducted an observational study using data from three waves (2011–15) of the China Health and Retirement Longitudinal Study, comprising 2609 participants with 6034 observations. IC was measured by five domains, including locomotion, cognition, psychological, sensory capacities, and vitality. We used joint latent class modelling to identify distinct classes with similar patterns of IC trajectory and onset of ADL disability, as well as to explore the variation in IC trajectory and predict five-year risks of ADL disability considering the heterogeneity in the elderly population.

**Results:**

The average baseline IC score was 7.15 (range: 0–15). We observed that IC scores slowly decreased with age, with 17.25% of participants developing ADL disability. We identified three classes of IC, which could be described as moderate health (class 1: n = 1634, 62.63%), at-risk (class 2: n = 716, 27.44%; had the highest risk of ADL disability), and optimal health (class 3: n = 259, 9.93%; had the lowest baseline risk of ADL disability). The probability of being in the moderate health class was decreased the most by emotional problems (odds ratio (OR) = 0.219; *P* < 0.001). Having a self-rated poor standard of living substantially reduced the chances of moderate (OR = 0.308; *P* = 0.001) and optimal health (OR = 0.110; *P* < 0.001).

**Conclusions:**

Observing IC trajectories and the onset of ADL disability can stratify the elderly into heterogeneous groups, as well as provide data for implementing person-centred care plans to reverse the trend and delay the adverse outcomes in clinical practice.

The trend of global population ageing has been accelerating in recent years, with the latest World Population Prospects reporting there are approximately 727.6 million ageing individuals (≥65 or older and above) worldwide, accounting for 9.33% of the world’s population [1]. This has had a significantly negative impact on health outcomes at the global level [2,3]. China is home to the largest ageing population globally [4], and has relatedly seen a rapid spread of disability in community-dwelling older adults, ranging from 23.7% to 28.6% [5]. This highlights a need for a more proactive, function-based approach to promoting optimal health of physical and mental ability for older adults [6,7]. In response to the challenges arising from global population ageing, the World Health Organization (WHO) proposed a novel framework for public health actions in 2015 [6]. This framework introduced the concept of intrinsic capacity (IC), designed as a composite measure of an individual's physical and mental capabilities [8] which is defined by five distinct domains – vitality, locomotion, sensory function, cognition, and psychological health [9].

Several measures of overall functioning in old age have been in use before the conceptualisation of IC, such as activities of daily living (ADL). However, ADL disability usually occurs only after functioning has substantially declined [10]. In contrast, the new WHO framework focusses on changes in capacity that may begin much earlier. Tracking these changes in relatively healthy individuals could allow for early interventions before significant deterioration in functioning [11]. Moreover, exploring the patterns of such changes and determinants thereof could help identify heterogeneity in the ageing process, enabling researchers and clinicians to provide more personalised care [11].

While studies have shown an association between IC and ADL, they have mostly been based on single IC measurements [11–13]. Stolz et al. [14] used a joint modelling approach to demonstrate that IC changes over time could dynamically predict the risk of ADL disability. However, their model did not address the heterogeneity in the elderly population. In this study, we sought to fill this gap by using joint latent class modelling (JLCM) to identify and examine the differences between distinct classes with similar patterns of IC trajectory and onset of ADL disability.

## METHODS

We reported our findings per the STROBE guidelines for a cross-sectional studies [15] (File S1 in the **Online Supplementary Document**).

### Data

We obtained our sample and data from the China Health and Retirement Longitudinal Study (CHARLS) [16,17], which collected information about the demographic background, health status and functioning, biomarkers, physical examination, health care, and insurance among a nationally representative sample of adults aged ≥45 years in China [16]. A total of 17 708 adults completed the baseline survey in 2011 (wave 1), with a response rate of 80.5%, and they completed the followed-up survey every 2–3 years (wave 2 in 2013, wave 3 in 2015). Here we used data from waves 1–3 data for analysis. We included participants who had IC values for at least one wave, and excluded those aged <60 or >80 years on their first participation; those with ADL disability at the time of first enrolment; and those with missing data of important independent variables (e.g. gender) during the first waves.

### Structure of the JLCM

JLCM is a type of joint modelling which can investigate the link between repeated measures of a longitudinal marker (in this case, IC) and an event of interest (occurrence of ADL disability) [18]. The joint model comprises three submodels: a linear mixed model to describe the trajectory of the longitudinal marker (IC); a survival model to determine the risk of the event (occurrence of ADL disability); and a multinomial logistic regression to model the latent class membership probability, which associates the longitudinal marker with the event of interest [18]. Each class has a class-specific marker trajectory and risk of event.

In JLCMs, non-Gaussian markers can be transformed by a link function into a Gaussian latent process with a mean intercept of 0 and variance of 1 [19], which can be then modelled through linear regression.

We modelled the probabilities of class membership using a multinomial logistic regression with gender and a classification based on the number of confirmed chronic diseases (classification of chronic diseases (CCD) – defined below). In addition, we formulated class-specific trajectories of the latent process as a linear mixed model, incorporating class-specific fixed effects of time, gender, and CCD interaction, along with interactions between gender and CCD, gender and age, and CCD and age. The model also accounted for random effects, including class-specific random intercepts and random effects of time. The risk of the event was modelled by a proportional hazards model with the covariates gender and CCD, while class-specific baseline hazard functions were estimated with piecewise constant hazards (three nodes).

### Longitudinal marker (IC)

The longitudinal marker is the outcome variable in the linear regression submodel of the JLCM, which, in the case of our study, was IC. We therefore included only IC observations at ages ≤80 in the model, as there were relatively few observations at ages >80, which could have interfered with model convergence.

IC was measured across five domains, as defined in the WHO’s 2017 guidelines on integrated care for older people [7]: locomotion (walking speed, the chair-stand test, balance); cognition (episodic memory and intact mental status); psychological (sleep quantity/quality, affect); sensory capacities (hearing and vision impairments); and vitality (grip strength, forced expiratory volume, haemoglobin). The five domains of IC were validated in the CHARLS cohort with good construct validity [20]. We converted the final extracted IC score into a scale of 0−15 (three scores for each domain), with higher scores indicating higher intrinsic capacity (Data S1 in the **Online Supplementary Document**).

### ADL survival outcome measures

The event of interest in the survival submodel of the JLCM was ADL disability. Here, ADL was measured with the following six items: do you have any difficulty with dressing; bathing or showering; eating; getting into or out of bed; using the toilet; controlling urination and defecation. Each item had four choices: ‘No, I don’t have any difficulty’ (scored as 3); ‘I have difficulty but can still do it’ (scored as 2); ‘Yes, I have difficulty and need help’ (scored as 1); ‘I cannot do it’ (scored as 0). ADL disability was defined as having difficulty in one or more items [21].

### Covariates

Age at each observation was recentred and scaled according to the formula *t* = (age −60)/10, to be used as the time variable in the model. The recentering sets the model intercepts at age 60 instead of age 0, which is more clinically relevant for the elderly cohort in this study. Additionally, scaling age by a factor of ten and expressing it in decades helps prevent overly small coefficient and variance estimates, improving the model's stability and reliability. However, we presented the figure axis labels in the original age scale in this manuscript to improve legibility.

CCD represents a classification based on the number of confirmed chronic diseases, whereby participants were classed as CCD = 0 in the case of zero confirmed chronic diseases; CCD = 1 if they had one confirmed chronic disease; and CCD = 2 when there were two or more confirmed chronic diseases.

### Statistical analysis

We conducted our analysis R, version 4.2.3 (R Core Team, Vienna, Austria), constructing the JLCM the ‘lcmm’ package [19]. *P*-values <0.05 denotes statistical significance.

We described continuous variables as means and standard deviations (SDs) and/or medians and interquartile ranges (IQRs), and categorical variables as frequencies and percentages. We compared groups either using analysis of variance (ANOVA) or the Wilcoxon test, or χ^2^ tests to examine between-group differences.

We approached the analysis in several steps. First, we needed to establish assumptions for the survival submodel, since time to ADL disability was interval-censored; however, the current implementation of JLCM in R could only handle left-truncated and right-censored data. We compared survival curves under three different sets of assumptions, that is, assuming the time of the event to be the earliest point, midpoint, or latest point of the interval. As the resulting survival curves were similar, we considered the interval midpoint as the time of the event in subsequent analysis.

Model selection was the second step of the analysis. We first fit four single-class JLCMs using combinations of two formulations of the linear mixed model (linear or quadratic terms of time) and two link functions (linear or beta). After selecting a formulation and link function, we fit models with two and three classes to determine the most suitable number of classes. We selected appropriate models based on the Aikake information criterion (AIC), the Bayesian information criterion (BIC), the mean posterior probabilities of class membership, and interpretability.

Having selected a model and identified the latent classes, the third step of our analysis was to investigate variations between classes. We first used the χ^2^ test and ANOVA test to compare characteristics separately, then used two multinomial regressions to compare characteristics in different aspects (i.e. morbidity and socio-economic factors). Finally, we performed an exploratory analysis of dynamic predictions using the JLCM. As existing literature usually used baseline characteristics for risk prediction, we chose a subset of participants with the same gender, CCD, and baseline IC; demonstrated the variation in their IC trajectory; and predicted five-year risks of ADL disability which were updated after each IC measurement.

## RESULTS

### Cohort description

**Figure 1:**
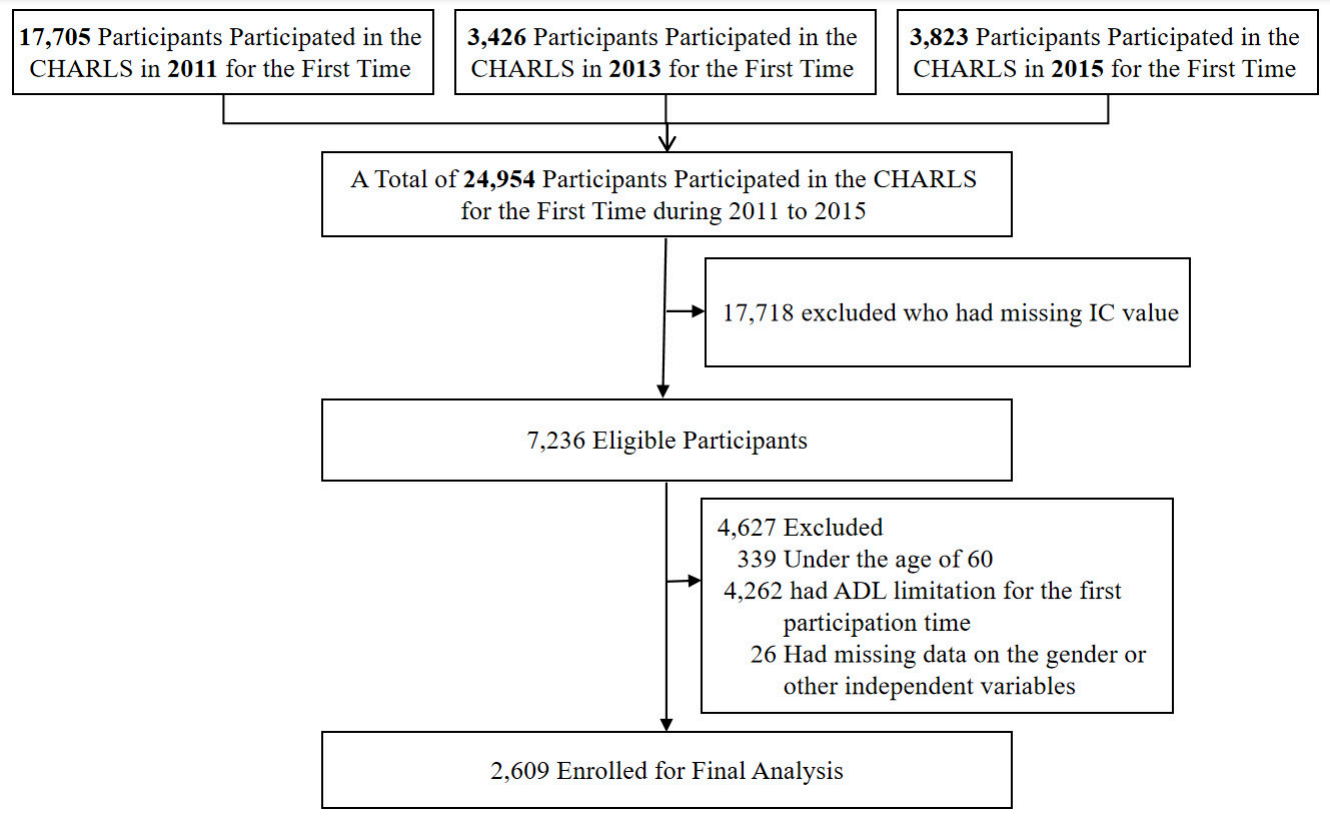
Participant flowchart.

Out of 24 954 participants enrolled in the CHARLS database, 2609 participants with 6034 observations over the three waves fit our inclusion criteria (**Figure 1**), with 1832 observations in the first wave (year 2011), 2058 in the second wave (year 2013), and 2144 in the third wave (year 2015).

In total, 64.1% of the participants were men, while the average age of participants in the first survey (year 2011) was 64.95 years (SD = 4.98). Arthritis was the most common chronic disease (31.0%), followed by hypertension (26.8%). Participants had an average of 1.41 (SD = 1.38) and a median of 1 (IQR = 0−2) confirmed chronic diseases; 30.6% had no confirmed chronic diseases, 29.8% had one, and 39.6% had two or above (**Table 1**).

**Table 1:** Baseline characteristics of study participants*

Variables	Overall (n = 2609)	Class 1 (n = 1634)	Class 2 (n = 716)	Class 3 (n = 259)	*P*-value
Age in years, x̄ (SD)	64.95 (4.98)	65.16 (5.17)	63.84 (4.11)	66.68 (5.35)	<0.001
Gender					<0.001
*Male*	1673 (64.1)	1095 (67.0)	386 (53.9)	192 (74.1)	
*Female*	936 (35.9)	539 (33.0)	330 (46.1)	67 (25.9)	
Marital status					0.548
*Married*	2194 (84.1)	1370 (83.9)	600 (83.8)	224 (86.5)	
*Unmarried*	414 (15.9)	263 (16.1)	116 (16.2)	35 (13.5)	
Number of multimorbidities					
*Hypertension*	698 (26.9)	402 (24.6)	276 (38.5)	20 (7.7)	<0.001
*Dyslipidaemia*	273 (10.5)	148 (9.1)	117 (16.3)	8 (3.1)	<0.001
*Diabetes*	158 (6.1)	86 (5.3)	68 (9.5)	4 (1.5)	<0.001
*Cancer*	23 (0.9)	13 (0.8)	10 (1.4)	0 (0.0)	0.099
*Chronic lung diseases*	309 (11.8)	159 (9.7)	149 (20.8)	1 (0.4)	<0.001
*Liver disease*	106 (4.1)	53 (3.2)	51 (7.1)	2 (0.8)	<0.001
*Heart disease*	347 (13.3)	181 (11.1)	151 (21.1)	15 (5.8)	<0.001
*Stroke*	53 (2.0)	19 (1.2)	32 (4.5)	2 (0.8)	<0.001
*Kidney disease*	152 (5.8)	69 (4.2)	80 (11.2)	3 (1.2)	<0.001
*Digestive disease*	559 (21.4)	287 (17.6)	257 (35.9)	15 (5.8)	<0.001
*Psychiatric problems*	31 (1.2)	12 (0.7)	19 (2.7)	0 (0.0)	<0.001
*Memory-related disease*	30 (1.1)	15 (0.9)	14 (2.0)	1 (0.4)	0.045
*Arthritis*	808 (31.0)	419 (25.6)	376 (52.5)	13 (5.0)	<0.001
*Asthma*	119 (4.6)	56 (3.4)	63 (8.8)	0 (0.0)	<0.001
BMI (kg/m^2^)					0.155
*<18.5*	154 (6.4)	90 (5.9)	53 (7.9)	11 (4.7)	
*18.5–25.0*	1600 (66.0)	1021 (67.3)	426 (63.3)	153 (64.8)	
*≥25.0*	671 (27.7)	405 (26.7)	194 (28.8)	72 (30.5)	
Smoking					0.061
*Nonsmoker or ex-smoker*	1323 (50.7)	856 (52.5)	338 (47.2)	129 (49.8)	
*Current smokers*	1284 (49.3)	776 (47.5)	378 (52.8)	130 (50.2)	
Drinking					0.001
*Non-drinker*	818 (31.4)	549 (33.6)	186 (26.0)	83 (32.0)	
*Ex-drinker*	213 (8.2)	129 (7.9)	55 (7.7)	29 (11.2)	
*Current drinkers*	1576 (60.5)	954 (58.5)	475 (66.3)	147 (56.8)	
Self-rated standard of living					<0.001
*Very high*	4 (0.2)	1 (0.1)	2 (0.3)	1 (0.4)	
*Relatively high*	76 (3.1)	51 (3.4)	7 (1.0)	18 (7.8)	
*Average*	1409 (58.2)	923 (60.8)	340 (50.7)	146 (62.9)	
*Relatively poor*	746 (30.8)	443 (29.2)	244 (36.4)	59 (25.4)	
*Poor*	185 (7.6)	99 (6.5)	78 (11.6)	8 (3.4)	
Health insurance					
*UEBMI*	390 (14.9)	260 (15.9)	59 (8.2)	71 (27.4)	<0.001
*URBMI*	135 (5.2)	84 (5.1)	33 (4.6)	18 (6.9)	0.344
*NRCMS*	1816 (69.6)	1121 (68.6)	562 (78.5)	133 (51.4)	<0.001
*Others†*	40 (1.5)	21 (1.3)	19 (2.7)	0 (0.0)	0.005
Pension insurance					0.301
*None*	2028 (82.0)	1260 (81.5)	575 (83.8)	193 (80.1)	
*Yes*	445 (18.0)	286 (18.5)	111 (16.2)	48 (19.9)	
Fall history	373 (14.6)	212 (13.2)	135 (19.2)	26 (10.2)	<0.001
CCD	1.41 (1.38)	1.17(1.19)	2.32 (1.45)	0.32 (0.73)	<0.001
*Group 1‡*	799 (30.6)	533 (32.6)	67 (9.4)	199 (76.8)	
*Group 2§*	777 (29.8)	605 (37.0)	125 (17.5)	47 (18.1)	
*Group 3‖*	1033 (39.6)	496 (30.4)	524 (73.2)	13 (5.0)	

BMI – body mass index, CCD – classification of chronic diseases, NRCMS – new rural cooperative medical scheme, SD – standard deviation, UEBMI – urban employee basic medical insurance, URBMI – urban resident basic medical insurance

*Presented as n (%) unless specified otherwise.

†Includes government health care and private medical insurance.

‡Group 1 consists of individuals with zero confirmed chronic diseases.

§Group 2 includes those with one confirmed chronic disease.

‖Group 3 comprises individuals with two or more confirmed chronic diseases.

The average and median baseline IC were 7.15 (SD = 2.16) and 7 (IQR = 6–9), respectively, while the average and median IC across all observations were 7.04 (SD = 2.14) and 7 (IQR = 6–9). The IC distribution in each wave of the survey was mostly symmetrical, and concentrated in the middle, with IC scores slowly decreasing with age (Figures S1 and S2 in the **Online Supplementary Document**).

A total of 450 (17.25%) participants developed ADL disability. Event times were assumed to be the midpoint of the interval, as there did not seem to be great differences (Figure S3 in the **Online Supplementary Document**).

### Trajectory classes

Among the single-class JLCMs, the formulation with linear terms and beta link function had the lowest AIC and BIC (Table S1 in the **Online Supplementary Document**). We then compared models with one to three classes using this formulation (Table S2 in the **Online Supplementary Document**) and found that the model with three classes had lower AIC, but higher BIC than the one with two classes.

Mean posterior probabilities for each class in the two models were all higher than 70%, which is typically considered adequate (Table S3 in the **Online Supplementary Document**). Considering the IC distribution described above, we believed the three-class model may be more appropriate, as it separates the population into a large class with average IC and two smaller classes with high and low IC respectively (Figure S4 in the **Online Supplementary Document**).

Predicted latent process values were within the 95% confidence interval (CI) bands of observed values, indicating a good model fit (Figure S5 in the **Online Supplementary Document**). The link function, which transforms latent process values to IC scores, was almost linear (Figure S6 in the **Online Supplementary Document**). Model coefficients for the three submodels are shown in Table S4 in the **Online Supplementary Document**.

**Figure 2:**
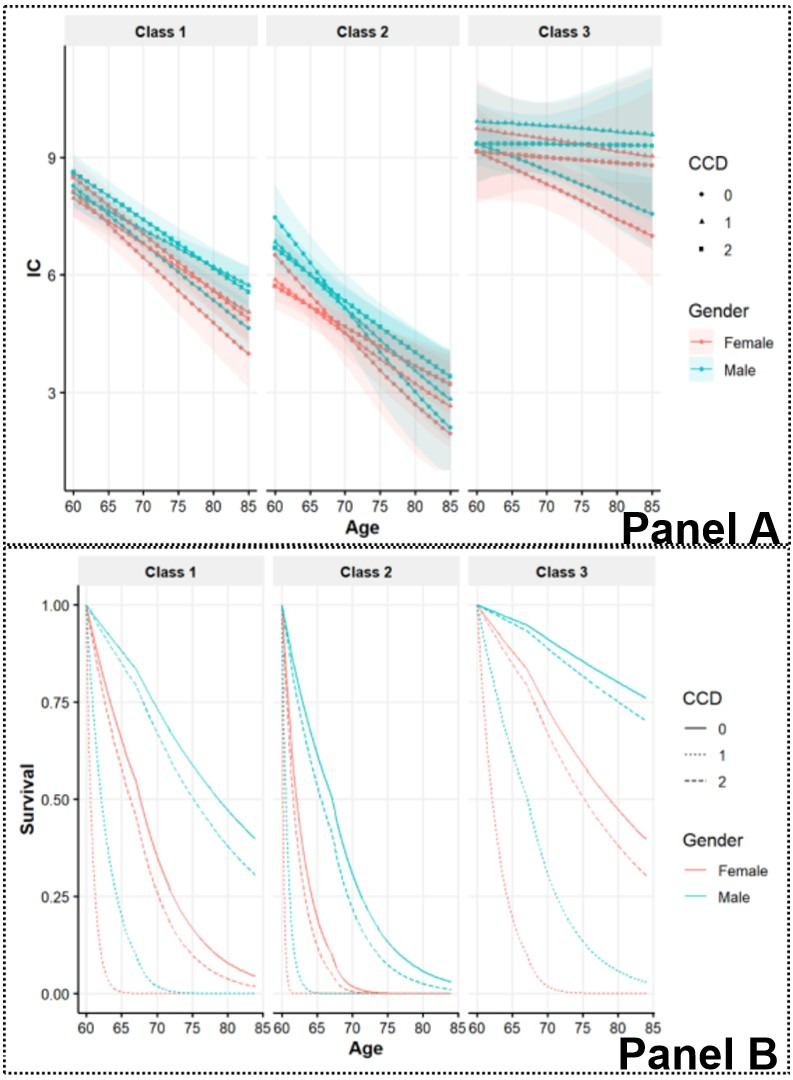
Model outcomes. **Panel A.** IC trajectory by class. **Panel B.** Probability of non-impaired ADL by class.

We then examined IC trajectories by class and probabilities of non-impaired ADL by class, as predicted by model coefficients (**Figure 2**). Class 1, which was the most populous class, showed moderate health. Class 3, the optimal health group, started with the highest IC and declined slower than the other classes. Class 2, the at-risk group, started with the lowest IC. Correspondingly, class 3 had the lowest baseline risk of ADL disability, while class 2 had the highest risk. A higher CCD significantly increased the probability of being in class 2 and class 1, while being female significantly increased the risk of ADL disability within each class.

**Figure 3:**
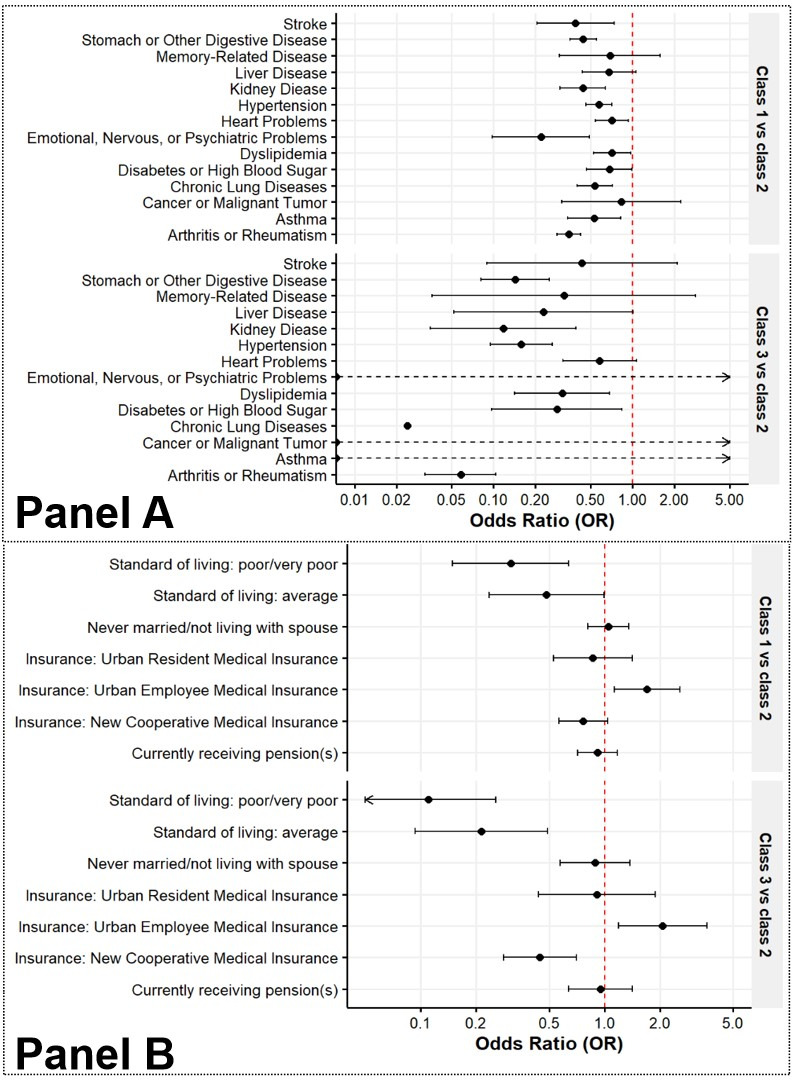
Forest plot of multinomial regression results. **Panel A.** Chronic diseases. **Panel B.** Socioeconomic factors.

We observed significant differences in most characteristics across the three classes (*P* < 0.05) (**Table 1**). Regression with morbidities showed that most chronic diseases were significantly associated with class membership. The probability of being in moderate health was decreased the most by emotional/nervous/psychiatric problems (OR = 0.219; 95% CI = 0.098–0.491, *P* < 0.001), followed by arthritis (OR = 0.347; 95% CI = 0.285–0.423, *P* < 0.001), stroke (OR = 0.388; 95% CI = 0.204–0.735, *P* = 0.004), and kidney disease (OR = 0.437; 95% CI = 0.300–0.636, *P* < 0.001). Class 3, meanwhile, had no subjects with asthma, psychiatric problems, or cancer, i.e. semi-complete separation. Aside from these diseases, participants with chronic lung diseases (OR = 0.024; 95% CI = 0.003–0.177, *P* < 0.001) and arthritis (OR = 0.058; 95% CI = 0.032–0.104, *P* < 0.001) had the least chance to be in optimal health (class 3) (**Figure 3**, Panel A; Table S5 in the **Online Supplementary Document** .

Among socioeconomic factors, Urban Employee Basic Medical Insurance (UEBMI) increased the probability of having moderate (OR = 1.702; 95% CI = 1.127–2.570, *P* = 0.012) and optimal health (OR = 2.066; 95% CI = 1.187–3.594, *P* = 0.010). Poor standards of living substantially reduced the chances of moderate (OR = 0.308; 95% CI = 0.149–0.635, *P* = 0.001) and optimal health (OR = 0.110; 95% CI = 0.047–0.256, *P* < 0.001) (**Figure 3**, Panel B; Figures S7 and S8 and Table S5 in the **Online Supplementary Document**).

### Dynamic predictions

**Figure 4:**
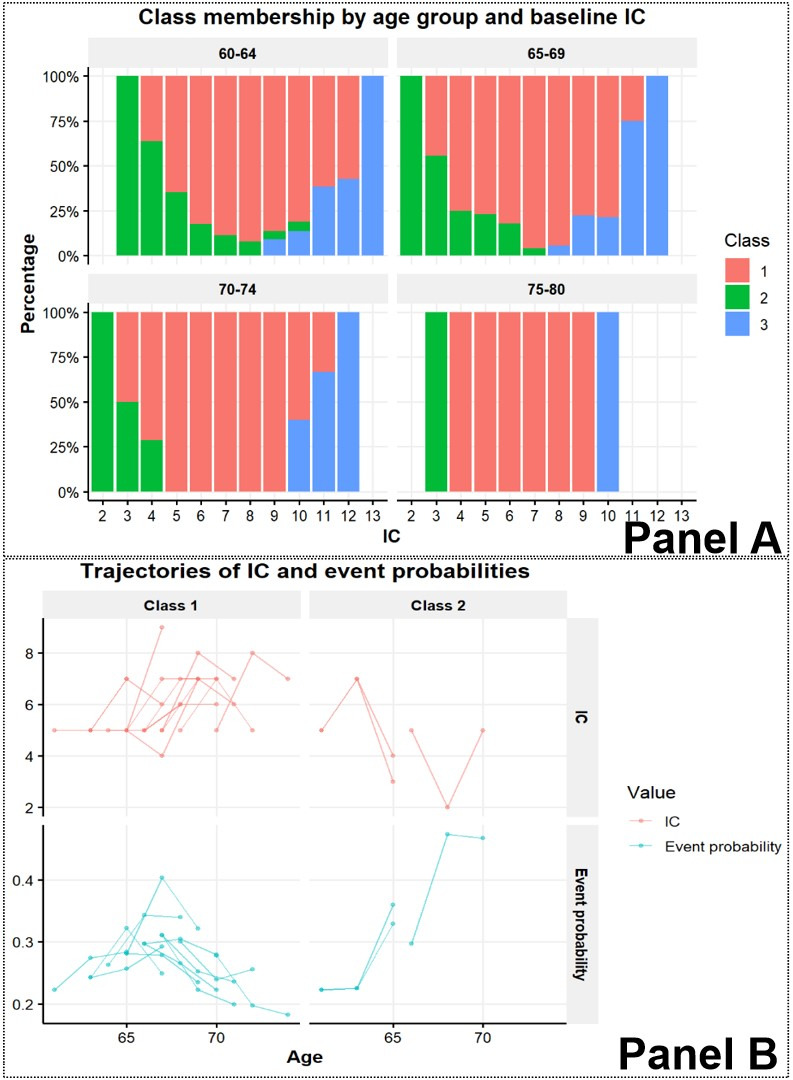
Risk prediction informed by IC trajectory information. **Panel A.** Class membership by baseline IC and age group. **Panel B.** IC trajectories and dynamic predictions of five-year risk of ADL disability.

The JLCM can use the information from multiple observations of IC to make more personalised risk predictions. As an example, we selected a subset of male participants with CCD = 1. The variation in predicted class membership among individuals with identical baseline characteristics (**Figure 4**, Panel A).

Dynamic predictions can be used to update individual risk predictions after each new IC measurement. To illustrate, we further selected participants from the above subset with the following conditions: baseline IC score of 5; three IC measurements available; last measurement made at or before the age of 74 years. **Figure 4**, Panel B shows the IC trajectories of this subgroup, and dynamic predictions of the risk of ADL disability within five years corresponding to each measurement. The predicted risks shift substantially, in accordance with the diverse IC trajectories, illustrating how new measurements refine individual risk assessments over time.

## DISCUSSION

Recent efforts to capture the notion of ‘successful ageing’ have shifted towards a function-based, rather than disease-centred approach [22,23]. Therefore, IC was conceived under this framework as a measure of overall health status to detect subtle changes in health in advance of significant functional losses [11,22]. Here we used a novel approach to explore the variability in IC trajectories and associated risk profiles of ADL disability. Based on longitudinal data of 2609 participants with 6034 observations from the nationally representative CHARLS database, we identified three subgroups characterised as moderate health, optimal health, and at-risk, which differed significantly across various characteristics. We also showed how repeated IC measurements over time can dynamically update individual risk predictions of ADL disability. Given the same basic starting conditions, continued monitoring of IC trajectories could help identify vulnerable elder individuals who may not have been detected by a single measurement, allowing subsequent targeted preventive interventions.

Stolz et al. [14] have previously used a joint model to characterise the association between IC trajectory and several negative health outcomes, including ADL disability, wherein they incorporated IC values as a covariate into the survival function. JLCM is also a type of joint model which aims to capture the entire association between marker and risk in the latent class structure without *a priori*-defined functions linking marker and risk [18]. Since a specific function was already given by Stolz et al. [14], there was no need to repeat this analysis. Instead, this study complements the previous work by analysing the heterogeneity of the population. Stolz et al. modelled an average IC trajectory and noted the considerable heterogeneity of IC trajectories between participants [14], emphasising the importance of accounting for this heterogeneity in further efforts to identify individuals in need of preventive care. The JLCM, meanwhile, is designed to address heterogeneous populations and uncover homogenous latent subgroups which make up the population [18]. The omission of a specific function between the two outcomes allows more flexibility in defining the baseline risk function [18].

Our analysis indicated that more than 60% of participants in our sample had moderate health, which is representative of the general state of older adults. Individuals in the moderate health group may experience common health issues such as chronic diseases and a gradual decline in physical function [24]. Their health status can be maintained or improved through health management, regular check-ups, and appropriate exercise [24,25]. Yildirim et al. [25] conducted a randomised controlled trial among 90 individuals aged ≥65 years and found that both aerobic exercise and a combination of aerobic and resistance exercises contributed to comparable improvements in strength, mobility, and flexibility. Aside from this, a good approach could be to use community and medical resources to provide necessary support and services for people with moderate health, such as health education and social activities [26].

Almost 30% of the participants in our sample were considered at-risk due to lower IC and a higher risk of ADL disability, indicating potential health concerns. Participants at risk may experience problems with falls, cognitive impairment, depression, sensory impairments, and muscle weakness due to lower IC. These problems can have adverse effects on their daily activities [27]. Communities or health care facilities can reduce the risk of disability by implementing early interventions, such as rehabilitation training, medication, and psychological support [28]. For example, Chen et al. [29] recruited 810 community-dwelling adults (age ≥50 years) and found that resilience played as a partial mediator between IC and happiness. Simultaneously, governments should pay more attention to high-risk groups when formulating public health policies and allocating resources to ensure that they have access to necessary medical and social support [30].

The smallest group (just under 10% of the sample) had optimal health, demonstrating higher IC and lower risk of ADL disability. Their health status should offers a learning opportunity in terms of healthy habits, lifestyle, and social support, which may contribute to maintaining good health [31]. People in optimal health groups should be encouraged to share their health experiences and lifestyles so that other older adults can benefit from them. Government and medical institutions should promote the dissemination of health knowledge and experience sharing by holding health lectures and exchange activities [32]. They could also develop individualised interventions based on the characteristics and needs of participants with different health conditions to promote the overall health and quality of life in older adults. Lastly, future research should focus on the health issues of the elderly to provide a scientific basis for future public health policy development. Communities or governments, in turn, should encourage optimal health groups to continue to maintain a healthy lifestyle by providing health incentives and regular health assessments [32]. 

Notably, emotional/nervous/psychiatric problems substantially increased the probability of being at-risk compared to other illnesses. However, there is much uncertainty in the OR estimate associated with this illness, as indicated by the wide CI. This could possibly be explained by the small number of participants with this illness, although it should be noted that such diseases may be underdiagnosed and public awareness may be low. For example, previous studies in China have found that only 10% of patients with general anxiety disorder [33] and 8% of those with major depressive disorder [34] were diagnosed. Further investigation may be needed to clarify whether the effect of emotional/nervous/psychiatric problems on health status is truly more pronounced than other diseases and whether caretakers and health care professionals need to pay special attention to the possibility of these problems.

Among socioeconomic factors, type of insurance was shown to impact health status classification, independent of living standards. New Rural Cooperative Medical Insurance (NRCMI) is part of China's universal health coverage system and can be considered a proxy for rural residents. Our results showed that participants with NRCMI may be disadvantaged. In recent years, China has been piloting the integration of this insurance model with the urban resident basic medical insurance (URBMI), which is also a part of the universal health coverage system. The aim of this project was to address health care inequities between rural and urban populations by pooling funding and aligning the reimbursement rate of the former with that of the latter [35–37]. However, as of the most recent CHARLS survey with IC measurements (year 2015), this integration program has only been piloted in four provinces and few participants were enrolled. It would be interesting to examine the impact of this integrated health insurance on IC and ADL with more recent data.

Some limitations of this study should be noted. First, we conducted a secondary analysis of data from the CHARLS longitudinal cohort, which was not designed with our study aims in mind, leading to limitations with important variables such as the IC. Second, although IC and its domains have been validated, there is yet no standardised approach to measuring and scoring this novel concept. Third, as the current implementation of JLCM in R could not handle interval-censored data, we made assumptions concerning the event time, which may have introduced bias. Fourth, our approach of using class membership predicted by the JLCM as the response variable in subsequent multinomial regressions does not account for the uncertainty of class membership predictions. To our knowledge, correction procedures addressing this issue have not yet been implemented in R. Furthermore, only three waves with IC measurements were available; this relatively small number of observations per person may explain why simple linear trajectories were found to be more appropriate than quadratic trajectories. It is possible that, as individual data spanning a longer time scale becomes available, more diverse patterns and trends may be discovered, providing a more comprehensive overview of the phenomenon.

## CONCLUSIONS

Through this study, we aimed to identify patterns in IC trajectory and the risk of ADL disability based on a large sample of 2609 participants from the nationally representative CHARLS database. We used a JLCM to uncover three subgroups that could be described as optimal health, moderate health, and at-risk. The model can also dynamically update the risk of ADL disability from data from repeated IC measurements. Geriatricians must measure IC domains and monitor natural trajectories of intrinsic capacity, allowing communities, hospitals, and governments to focus on the demands at the individual and health system levels and enabling them to implement early targeted interventions to minimise the adverse outcomes and ease public health burden.

## Additional material


Online Supplementary Document
