# Supplementary material for: Patterns of intrinsic capacity trajectory and onset of activities of daily living disability among community-dwelling older adults
Source: J Glob Health. 2024 Oct 11;14:04159. doi: 10.7189/jogh.14.04159 (PMC11467824; doi:10.7189/jogh.14.04159)
Supplement: Online Supplementary Document [file jogh-14-04159-s001.pdf]

Online Supplementary Document

Supplementary File 1. STROBE Checklist 3

Supplementary Data 1. Five Domain Measures of Intrinsic Capacity 4

Supplementary Figure 1. Distribution of intrinsic capacity scores in each wave of the survey 5

Supplementary Figure 2. Distribution of intrinsic capacity scores by age 6

Supplementary Figure 3. Survival curves under different assumptions 7

Supplementary Figure 4. Class N and Intrinsic Capacity 8

Supplementary Figure 5. Latent process predictions *vs.* Observations 9

Supplementary Figure 6. Link function 10

Supplementary Figure 7. Multinomial regression results with class 1 as reference 9

Supplementary Figure 8. Multinomial regression results with class 3 as reference 10

Supplementary Table 1. Selection of linear regression submodel and link function formulation 11

Supplementary Table 2. Selection of models with different numbers of latent classes 11

Supplementary Table 3. Mean posterior probabilities 11

Supplementary Table 4. Model coefficient in three different types of model 12

Supplementary Table 5. Multinomial regression coefficients 13

Supplementary file S1. STROBE Statement—Checklist of items that should be included in reports of cross-sectional studies

	Item No	Recommendation	Page
**Title and abstract**	1	(*a*) Indicate the study's design with a commonly used term in the title or the abstract	1
		(*b*) Provide in the abstract an informative and balanced summary of what was done and what was found	1-2
**Introduction**			
Background/rationale	2	Explain the scientific background and rationale for the investigation being reported	2-3
Objectives	3	State specific objectives, including any prespecified hypotheses	3
**Methods**			
Study design	4	Present key elements of study design early in the paper	3
Setting	5	Describe the setting, locations, and relevant dates, including periods of recruitment, exposure, follow-up, and data collection	3
Participants	6	(*a*) Give the eligibility criteria, and the sources and methods of selection of participants	3
Variables	7	Clearly define all outcomes, exposures, predictors, potential confounders, and effect modifiers. Give diagnostic criteria, if applicable	4-5
Data sources/ measurement	8*	For each variable of interest, give sources of data and details of methods of assessment (measurement). Describe comparability of assessment methods if there is more than one group	4-5
Bias	9	Describe any efforts to address potential sources of bias	5-6
Study size	10	Explain how the study size was arrived at	3
Quantitative variables	11	Explain how quantitative variables were handled in the analyses. If applicable, describe which groupings were chosen and why	4-5
Statistical methods	12	(*a*) Describe all statistical methods, including those used to control for confounding	5-6
		(*b*) Describe any methods used to examine subgroups and interactions	6
		(*c*) Explain how missing data were addressed	3
		(*d*) If applicable, describe analytical methods taking account of sampling strategy	5-6
		(*e*) Describe any sensitivity analyses	6
**Results**			
Participants	13*	(a) Report numbers of individuals at each stage of study—eg numbers potentially eligible, examined for eligibility, confirmed eligible, included in the study, completing follow-up, and analysed	6
		(b) Give reasons for non-participation at each stage	Figure 1
		(c) Consider use of a flow diagram	Figure 1
Descriptive data	14*	(a) Give characteristics of study participants (eg demographic, clinical, social) and information on exposures and potential confounders	6-7
		(b) Indicate number of participants with missing data for each variable of interest	6-7
Outcome data	15*	Report numbers of outcome events or summary measures	7
Main results	16	(*a*) Give unadjusted estimates and, if applicable, confounder-adjusted estimates and their precision (eg, 95% confidence interval). Make clear which confounders were adjusted for and why they were included	7-9
		(*b*) Report category boundaries when continuous variables were categorized	5
		(*c*) If relevant, consider translating estimates of relative risk into absolute risk for	-

		a meaningful time period	
Other analyses	17	Report other analyses done—eg analyses of subgroups and interactions, and sensitivity analyses	9
**Discussion**			
Key results	18	Summarise key results with reference to study objectives	9-10
Limitations	19	Discuss limitations of the study, taking into account sources of potential bias or imprecision. Discuss both direction and magnitude of any potential bias	12
Interpretation	20	Give a cautious overall interpretation of results considering objectives, limitations, multiplicity of analyses, results from similar studies, and other relevant evidence	10-12
Generalisability	21	Discuss the generalisability (external validity) of the study results	11-12
**Other information**			
Funding	22	Give the source of funding and the role of the funders for the present study and, if applicable, for the original study on which the present article is based	13

Supplementary Data 1. Five Domain Measures of Intrinsic Capacity

Locomotion

Locomotion capacity was measured by the Short Physical Performance Battery Test, including three tests: (1) walking speed test: participants were asked to walk 2.5 meters twice and the time (seconds) for both walks was recorded, the average 2.5-meters walking speed was scored one if it was greater than ≥ 1 meter/second. (2) chair-stand test: participants were asked to fold arms across the chest with five timed, repetitive chairs to a full stand-up, the time (seconds) for 5 repeats was recorded and ≤ 12 seconds scored 1 point. (3) balance test: (a)semi-tandem stand for at least 10 seconds, (b)full-tandem stand for at least 30 seconds for those aged 70 and above, or at least 60 seconds for those aged below 70, (c) side-by-side stand for at least 10 seconds, completion of two or more items is scored 1 point. Assign points of each test was from 0 (worst) to 1 point (best), higher scores indicate better physical performance (range 0-3).

Cognition

Cognition was measured by two parts: episodic memory and intact mental status. Episodic memory was based on the delayed recall scores (range 0-10). The higher the score, the better the performance. Intact mental status was measured mainly by the Telephone Interview of Cognitive Status (TICS) battery[1], including: (1) Serial 7 test (math): participators were asked to do subtraction of numbers that subtract 7 from 100 (up to five times, range 0-5). (2) Memory: participators should identify the current day, month, year, and season (range 0-4). (3) Draw: Participants were asked to draw the picture shown by the interviewer (range 0-1). The total scores of cognition w**ere** 0-20, and finally, cognition was classified into four groups, normal cognitive function (range 18-20, scored 3 points), mild cognitive dysfunction (range 14-17, scored 2

points), moderate cognitive dysfunction (range 7-13, scored 1 point) and severe cognitive dysfunction (range 0-6, scored 0 points).

Psychosocial

Psychosocial were measured by affect, sleep length, and quality. Affect was evaluated by the ten-item Center for Epidemiological Studies-Depression (CES-D) scale[2], except for the sleep dimension. The nine items recorded how the participant felt and behaved during the last week. Four categorized responses in each item, rarely or none of the time (<1 day), some or a little of the time (1-2 days), occasionally or a moderate amount of the time (3-4 days), most or all of the time (5-7 days). A total of nine items' scores were calculated by adding all responses (range 0-27). A total score of 0-9 was defined as no depression and was assigned 1 point. For sleep length, participants were asked 'During the past month, how many hours of actual sleep did you get at night (average hours for one night)?' and 'During the past month, how long (minutes) did you take a nap after lunch?', the total sleep length between 5 and 10.5 hours indicated good sleep length and was assigned 1 point. Sleep quality was assessed by reporting the frequency of restless sleep during the last week, and the total number of days between 0 and 2 was assigned 1 point.

Sensory

Three self-report items were used for assessing audition and vision impairments.

For the audition, participants were asked 'Is your hearing very good, good, fair, poor, or very poor', and selected any of the first 3 options and assigned 1 point. Vision status was measured by asking participants 'How good is your eyesight for seeing things at a distance, like recognizing a friend from across the street' and 'How good is your eyesight for seeing things up close, like reading ordinary newspaper print'. The choices included excellent, very good, good, fair, and poor, and participants selected

any of the first 3 options for each vision question and assigned 1 point.

Vitality

Vitality was measured by three indicators: (1) grip strength (kg): participants performed two measurements for each hand using a dynamometer (YueJian brand, Model WL-1000, Nantong Yuejian Physical Testing Equipment Company Limited), and the average value of the dominant hand was recorded for analysis (the larger measure was used if both hands were reported as dominant), the cut-off points of grip strength was ≥35kg for men and ≥25kg for women to show a better status and assigned 1 point; (2) forced expiratory volume (FEV): participants were asked to blow into the spirometer (Aipu peak flow meter, Shanghai Aipu Medical Equipment Plastic Company with Limited Liability) as hard as they could and as fast as possible for a total of 3 measurements, and the most satisfactory FEV measurements were recorded for data analysis, we used cut-off points of ≥400 for men and ≥290 for women to show a better FEV and assigned 1 point; (3) hemoglobin: hemoglobin value was obtained from the blood dataset.

Reference

1 *Lei X, Smith JP, Sun X, Zhao Y. Gender differences in cognition in china and reasons for change over time: evidence from charls. J Econ Ageing. 2014;4:46-55.*

2 *Carleton RN, Thibodeau MA, Teale MJ, Welch PG, Abrams MP, Robinson T, et al. The center for epidemiologic studies depression scale: a review with a theoretical and empirical examination of item content and factor structure. Plos One. 2013;8(3):e58067.*

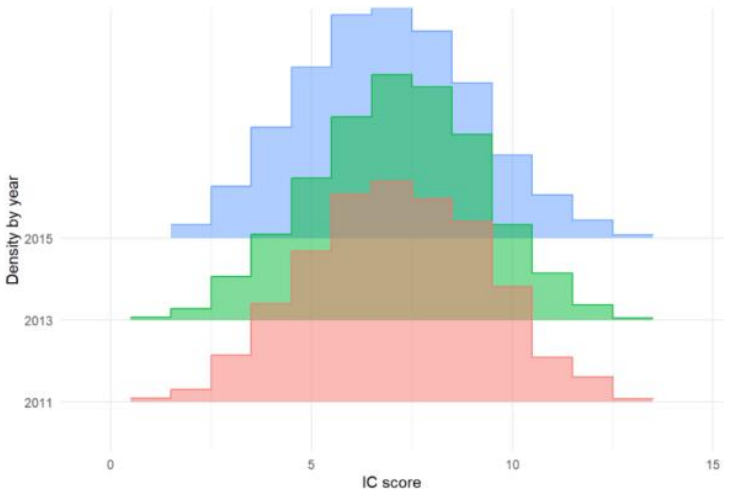

Supplementary Figure 1. Distribution of intrinsic capacity scores in each wave of the survey

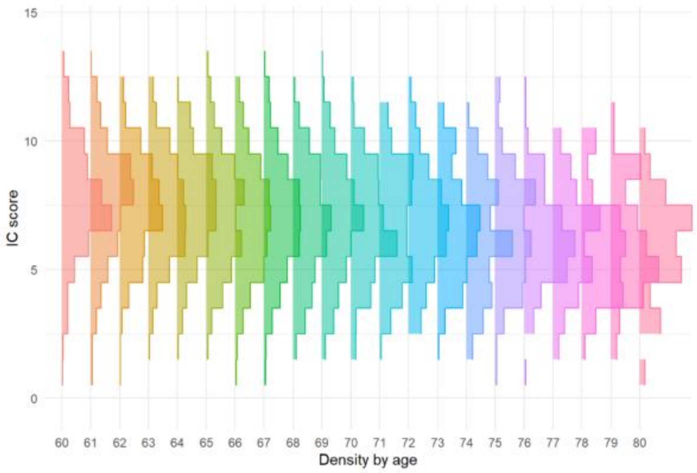

Supplementary Figure 2. Distribution of intrinsic capacity scores by age

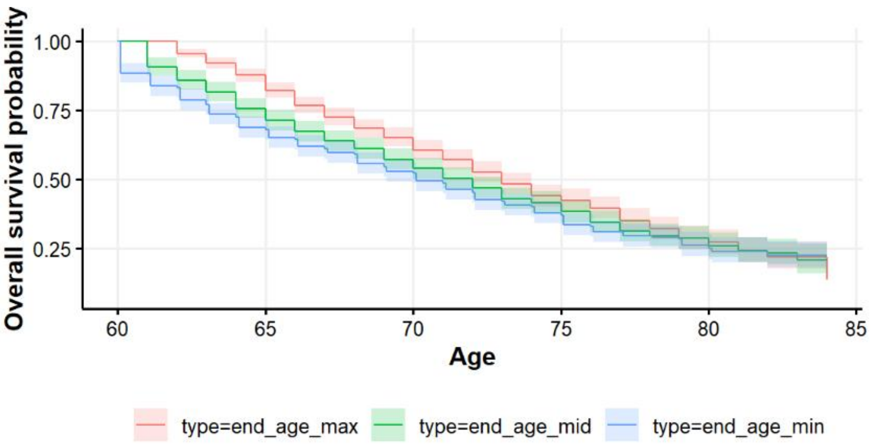

Supplementary Figure 3. Survival curves under different assumptions

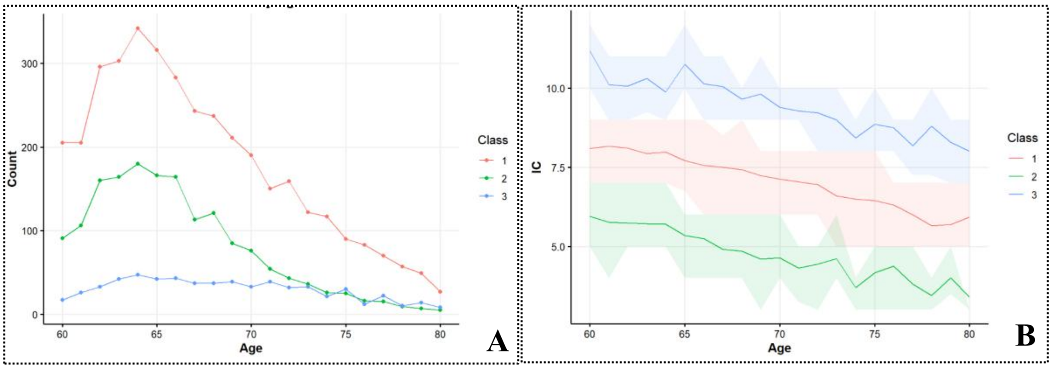

Supplementary Figure 4. Class N and Intrinsic Capacity

Note: A: Class N by age; B: Intrinsic capacity by class and age.

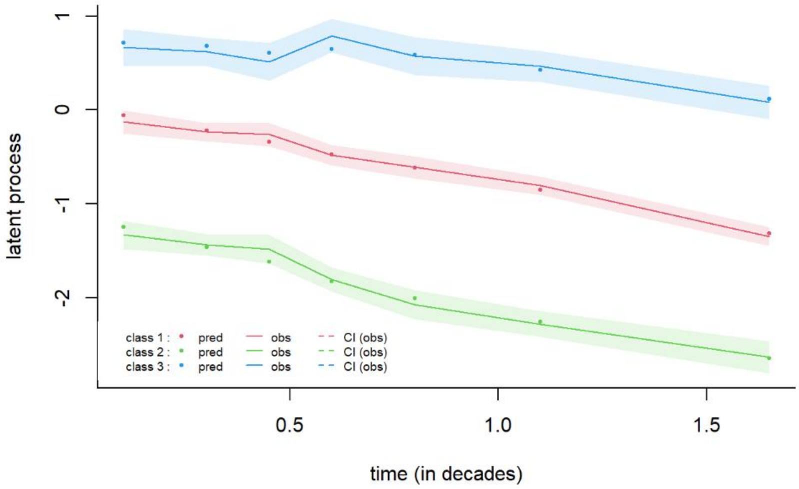

Supplementary Figure 5. Latent process predictions *vs.* Observations

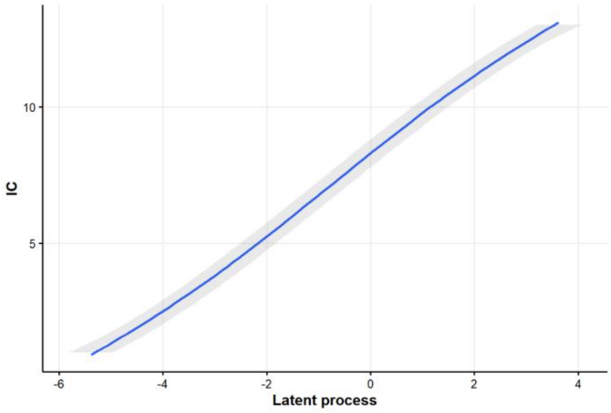

Supplementary Figure 6. Link function

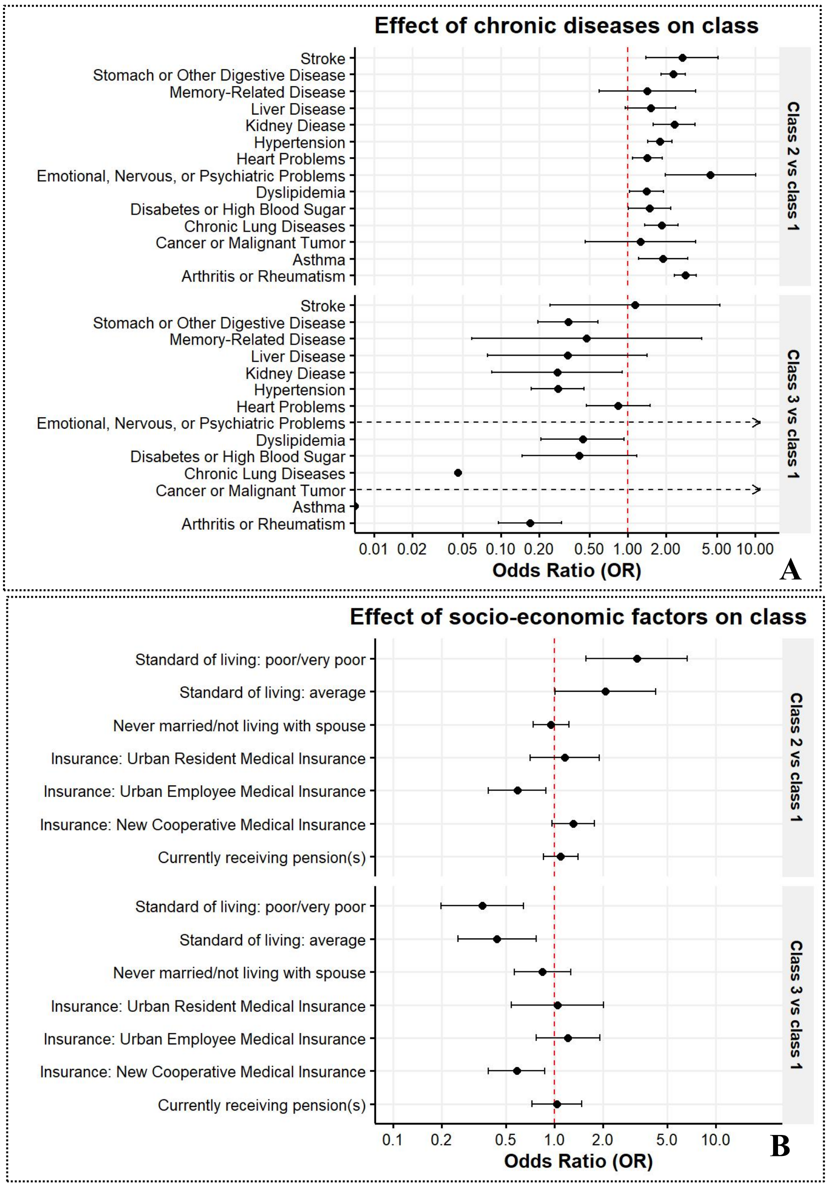

Supplementary Figure 7. Multinomial regression results with class 1 as reference

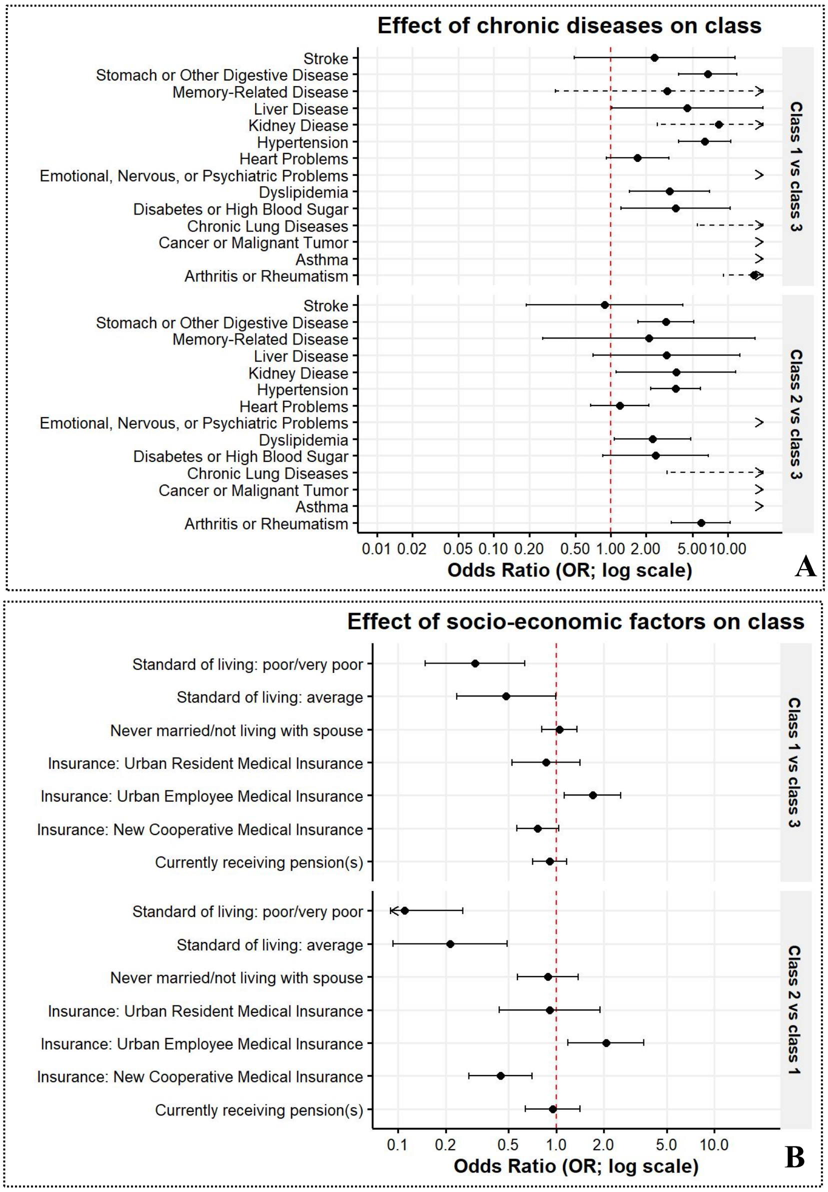

Supplementary Figure 8. Multinomial regression results with class 3 as reference

Supplementary Table 1. Selection of linear regression submodel and link function formulation

	Formula	Link function	AIC	BIC
Submodel 1	Linear	Linear	26054.87	26154.61
Submodel 2	Quadratic	Linear	26045.90	26169.10
**Submodel 3**	**Linear**	**Beta**	**26021.06**	**26132.53**
Submodel 4	Quadratic	Beta	26011.74	26146.67

Supplementary Table 2. Selection of models with different numbers of latent classes

	The numbers of latent class	AIC	BIC	%class1	%class2	%class3
Model 1	1	26021.06	26132.53	100.00	-	-
Model 2	2	25922.38	26110.11	36.76	63.24	-
Model 3	3	25915.12	26179.12	62.63	27.44	9.93

Supplementary Table 3. Mean posterior probabilities

	class1	class2	class3
**Model 2 (two latent classes)**			
N	959	1650	-
%	36.76	63.24	-
prob1	**0.786**	0.2059	-
prob2	0.214	**0.7941**	-
**Model 3 (three latent classes)**			
N	1634	716	259
%	62.63	27.44	9.93
prob1	**0.7523**	0.2000	0.2607
prob2	0.1395	**0.7967**	0.0057
prob3	0.1082	0.0033	**0.7337**

Note:
N: Number of subjects in each class;
prob1: Mean posterior probability of membership in class 1 among subjects in each class;
prob2: Mean posterior probability of membership in class 2 among subjects in each class;
prob3: Mean posterior probability of membership in class 3 among subjects in each class.

Supplementary Table 4. Model coefficient in three different types of model

	Submodel	Coefficient	Value	95%*CI* lower	upper	*P*-value
1	CMM	intercept class 3 (reference class)				
2	CMM	intercept class 2	1.838	0.913	3.700	0.088
3	CMM	intercept class 1	0.638	0.233	1.743	0.381
4	CMM	female class1	1.225	0.539	2.787	0.628
5	CMM	female class2	1.865	0.712	4.881	0.204
6	CMM	ci1 class1	**4.492**	1.769	11.404	0.002
7	CMM	ci1 class2	**5.176**	1.485	18.036	0.010
8	CMM	ci2 class1	**6.115**	1.800	20.768	0.004
9	CMM	ci2 class2	**27.741**	6.646	115.795	<0.001
10	PHM	class 3 PH (reference class)				
11	PHM	class 1 PH	3.369	1.155	9.826	0.026
12	PHM	class 2 PH	12.773	4.583	35.600	<0.001
13	PHM	female	1.291	1.011	1.649	0.041
14	PHM	ci1	0.882	0.574	1.356	0.568
15	PHM	ci2	1.051	0.698	1.582	0.811
16	LMM	intercept class 1 (reference class)				
17	LMM	intercept class2	-0.542	-1.455	0.556	0.110
18	LMM	intercept class3	0.760	0.191	1.328	0.009
19	LMM	age_*tf* class1	**-0.988**	-1.234	-0.743	<0.001
20	LMM	age_*tf* class2	**-1.561**	-2.281	-0.842	<0.001
21	LMM	age_*tf* class3	**-0.500**	-0.835	-0.166	0.003
22	LMM	female class1	-0.095	-0.618	1.025	0.568
23	LMM	female class2	**-0.653**	-0.339	1.585	<0.001
24	LMM	female class3	-0.168	-0.925	0.589	0.663
25	LMM	ci1 class1	-0.118	0.571	2.434	0.574
26	LMM	ci1 class2	-0.421	0.396	2.892	0.179
27	LMM	ci1 class3	0.407	-0.350	1.164	0.292
28	LMM	ci2 class1	0.241	0.588	3.033	0.290
29	LMM	ci2 class2	-0.527	1.894	4.752	0.052
30	LMM	ci2 class3	-0.006	-0.588	0.577	0.985
31	LMM	age_*tf**female class1	-0.149	-0.400	0.102	0.245
32	LMM	age_*tf**female class2	0.204	-0.155	0.562	0.266
33	LMM	age_*tf**female class3	-0.074	-0.67	0.522	0.808
34	LMM	age_*tf**ci1 class1	0.352	0.048	0.657	0.023
35	LMM	age_*tf**ci1 class2	0.429	-0.362	1.220	0.288
36	LMM	age_*tf**ci1 class3	0.387	-0.369	1.142	0.316
37	LMM	age_*tf**ci2 class1	0.160	-0.147	0.468	0.307
38	LMM	age_*tf**ci2 class2	0.643	-0.066	1.351	0.076
39	LMM	age_*tf**ci2 class3	0.469	-0.161	1.098	0.144

Note: It should be noted that coefficients in the linear mixed model are in the latent process scale. Coefficients without CCD and p-values were fixed by the algorithm for identifiability.
CMM: class membership model (multinomial logistic regression);
PHM: proportional hazards model;
LMM: linear mixed models;
ci 1: one chronic disease;
ci 2: two or more chronic diseases;
age_*tf*: (age-60)/10.

Supplementary Table 5. Multinomial regression coefficients

Variables	Class 1 *OR*	Class 1 lower *CI*	Class 1 upper *CI*	Class 1 *P* value	Class 3 *OR*	Class 3 lower *CI*	Class 3 upper *CI*	Class 3 *P* value
**Chronic diseases**								
Intercept	7.397	6.230	8.782	<0.001	2.641	2.123	3.285	<0.001
Hypertension	0.571	0.460	0.708	<0.001	0.158	0.095	0.264	<0.001
Dyslipidemia	0.711	0.523	0.965	0.029	0.311	0.142	0.683	0.004
Diabetes	0.679	0.465	0.991	0.045	0.285	0.097	0.836	0.022
Cancer	0.829	0.309	2.225	0.709	-	-	-	-
Chronic lung diseases	0.534	0.399	0.714	<0.001	0.024	0.003	0.177	<0.001
Liver disease	0.677	0.433	1.057	0.086	0.228	0.052	1.005	0.051
Heart disease	0.708	0.540	0.927	0.012	0.578	0.315	1.062	0.077
Stroke	0.388	0.204	0.735	0.004	0.432	0.089	2.089	0.297
Kidney disease	0.437	0.300	0.636	<0.001	0.117	0.035	0.392	0.001
Digestive disease	0.440	0.353	0.547	<0.001	0.143	0.081	0.252	<0.001
Psychiatric problems	0.219	0.098	0.491	<0.001	-	-	-	-
Memory-related disease	0.686	0.297	1.584	0.378	0.321	0.036	2.832	0.307
Arthritis	0.347	0.285	0.423	<0.001	0.058	0.032	0.104	<0.001
Asthma	0.529	0.341	0.821	0.004	-	-	-	-
**Socio-economic factors**								
Intercept	6.901	3.168	15.03	<0.001	3.263	1.287	8.272	0.013
UEBMI	1.702	1.127	2.570	0.012	2.066	1.187	3.594	0.010
URBMI	0.864	0.527	1.417	0.564	0.907	0.436	1.889	0.794
NRCMS	0.763	0.563	1.036	0.083	0.445	0.282	0.702	0.001
Average-living	0.483	0.235	0.994	0.048	0.213	0.093	0.488	<0.001
Poor-living	0.308	0.149	0.635	0.001	0.110	0.047	0.256	<0.001
Pension insurance	0.914	0.713	1.170	0.475	0.950	0.639	1.411	0.799
Unmarried	1.049	0.813	1.353	0.713	0.886	0.572	1.373	0.589

Note:
UEBMI, Urban Employee Basic Medical Insurance;
URBMI, Urban Resident Basic Medical Insurance;
NRCMS, New Rural Cooperative Medical Scheme;
Average-living, Self-rated standard of living=Average;
Poor-living, Self-rated standard of living=Relatively poor or Poor;
Pension insurance, Currently receiving at least one kind of pension.

References

1 Lei X, Smith JP, Sun X, Zhao Y. Gender differences in cognition in china and reasons for change over time: evidence from charls. J Econ Ageing. 2014;4:46-55.

2 Carleton RN, Thibodeau MA, Teale MJ, Welch PG, Abrams MP, Robinson T, et al. The center for epidemiologic studies depression scale: a review with a theoretical and empirical examination of item content and factor structure. Plos One. 2013;8(3):e58067.
